# The protective effect of biologic and targeted-synthetic therapies on developing multisystem inflammatory syndrome in children

**DOI:** 10.3389/fped.2025.1607637

**Published:** 2025-07-18

**Authors:** Lana Khoury, Adi Miller-Barmak, Shereen Shehadeh, Hilla Cohen, Dana Hadar, Mohamad Hamad Saied

**Affiliations:** ^1^ Department of Pediatrics, Carmel Medical Center, Haifa, Israel; ^2^ Pediatric Rheumatology Service, Ruth Rappaport Children's Hospital, Rambam Health Care Campus, Haifa, Israel; ^3^ Pediatric Infectious Unit, Carmel Medical Center, Haifa, Israel; ^4^ Rappaport Faculty of Medicine, Technion-Israel Institute of Technology, Haifa, Israel; ^5^ Research Authority, Clalit Health Care Organization, Carmel Medical Center, Haifa, Israel; ^6^ Department of Pediatric Immunology and Rheumatology, Wilhelmina Children's Hospital, University Medical Center Utrecht, Utrecht, Netherlands

**Keywords:** MIS-C, COVID-19, biological treatments, pediatric, targeted-synthetic therapies

## Abstract

**Background:**

Multisystem Inflammatory Syndrome in Children (MIS-C) is a severe, life threatening, complication that arises weeks after acute Coronavirus disease 2019 (COVID-19) infection, often presenting with fever and diverse systemic symptoms. Limited data exists on the effectiveness of biologic and targeted-synthetic therapies in preventing MIS-C development. Therefore, our aim was to investigate whether biologic and targeted-synthetic therapies can prevent the occurrence of MIS-C.

**Methods:**

We assessed the Clalit Health Services database, the largest health care organization in Israel, data from 793,909 children aged 0–18 years who tested positive for COVID-19 were analyzed. The diagnosis of MIS-C was adjudicated using the case definition used by the Centers for Disease Control and Prevention (CDC) or by the World Health Organization (WHO). Patients receiving biologic and targeted-synthetic therapies were compared to a control group.

**Results:**

Among 793,909 cases, 573 children received biologic and targeted-synthetic therapies, and 143 cases of MIS-C were identified. Notably, none of the individuals treated with biologic and targeted-synthetic therapies developed MIS-C.

**Conclusion:**

Our study highlights our hypothesis on the efficacy of biological treatments in preventing MIS-C. Although statistical significance was not achieved due to the absence of MIS-C cases in patients receiving biologic and targeted-synthetic therapies, our study shows a possible association between biological therapies and reduced risk of MIS-C following COVID-19 infection in children. Further research, including prospective studies with larger cohorts, is warranted to confirm these findings and elucidate underlying mechanisms.

## Background

Multisystem Inflammatory Syndrome in Children (MIS-C) is a systemic inflammatory syndrome that occurs 2–6 weeks after acute Coronavirus disease 2019 (COVID-19) infection (1). Clinical presentation typically includes fever and often gastrointestinal or cardiac symptoms but can involve other body systems as well (2). Additionally, many children present with symptoms resembling Kawasaki-like disease such as conjunctival injection, cervical lymphadenopathy, and appropriate skin involvement (3). Most patients with MIS-C require hospitalization for aggressive management due to significant cardiac involvement, which may lead to cardiogenic shock, and approximately 2% of them succumb to this condition (4, 5).

Two diagnostic criteria for MIS-C are accepted worldwide: the criteria from the World Health Organization (WHO) and those from the Centers for Disease Control and Prevention (CDC) (6, 7). According to the CDC, patients up to the age of 21 are included in the criteria, whereas the WHO includes patients only up to the age of 19.

The pathogenesis of MIS-C involves dysregulation of various proinflammatory cytokines including IL-1, IL-6, IL-18, TNF-α, and IFN-γ, leading to cytokine storm and widespread systemic inflammation (8).

The first line of treatment for MIS-C includes steroids and intravenous immunoglobulin (IVIG) along with anti-thrombotic and anticoagulant therapies (9). Children who do not respond to these treatments receive different biological therapies such as anti-TNF or anti-IL1 and anti- IL6 (10).

To date, there is limited data on the proportion of children receiving biologic and targeted-synthetic therapies who had developed MIS-C. In a cohort of 55 children with rheumatic diseases who had COVID-19, 31 of them were treated with biologic and targeted-synthetic therapies, there were no cases of MIS-C (11). Conversely, in a study with a cohort of 26 rheumatologic patients treated with biological therapies who had COVID-19 infection, 5 of them (19.2%) developed MIS-C (12). In another study with a cohort of 113 children who had COVID-19 and received biological treatments, the MIS-C rate was 4.4% (13).

In this “big data” study we assessed the Clalit medical database in a cohort study to compare the percentage of children and adolescents up to the age of 18 who were treated with biologic and targeted-synthetic therapies and developed MIS-C after contracting COVID-19, vs. the percentage of individuals in the same age group who did not receive biological therapy, contracted COVID-19, and developed MIS-C in order to evaluate whether biologic and targeted-synthetic therapies may have a protective effect from the devastating MIS-C.

## Methods

The study is a retrospective cohort study based on the Clalit health service database, represent the major Israeli health organization and one of the largest health service organizations in world, delivering health care services to about 5,000,000 insured subjects, highly computerized and continuously updated.

This study included children and adolescents aged 0–18 who contracted COVID-19 between March 2020 and February 2023, examining whether these patients developed MIS-C according to the CDC or WHO diagnostic criteria. The research group comprised patients who had received biologic and targeted-synthetic therapies within 31 days before their positive COVID-19 test date, compared to the control group who had not received such treatments. This timeframe was chosen because all biologic and targeted-synthetic therapies included in the study are typically administered on a monthly or more frequent schedule in pediatric clinical practice. Positive COVID-19 cases were determined based on either a positive RT-PCR test or a positive COVID-19 antigen test.

Our primary outcome was the percentage of COVID-19 patients who developed MIS-C in both groups. Secondary outcomes included demographic data (gender, age, origin), data regarding underlying diseases and biological treatment, data regarding COVID-19 infection, and data regarding MIS-C.

The biologic and targeted-synthetic therapies included in this study were the biological agents in use among children and adolescents including Adalimumab, Etanercept, Golimumab, Infliximab, Tocilizumab, Canakinumab, Anakinra, Tofacitinib, Baricitinib, Ustekinumab, Abatacept, Rituximab, Upadacitinib, Secukinumab.

### Statistical analysis

Categorical variables were compared using Fisher’s exact test or chi-square, as appropriate, while continuous variables were compared using the two-sample Wilcoxon test. The analysis was performed using R software (version 4.3.1) from the R Foundation for Statistical Computing.

## Results

### Basic characteristics of the study population

**Figure 1:**
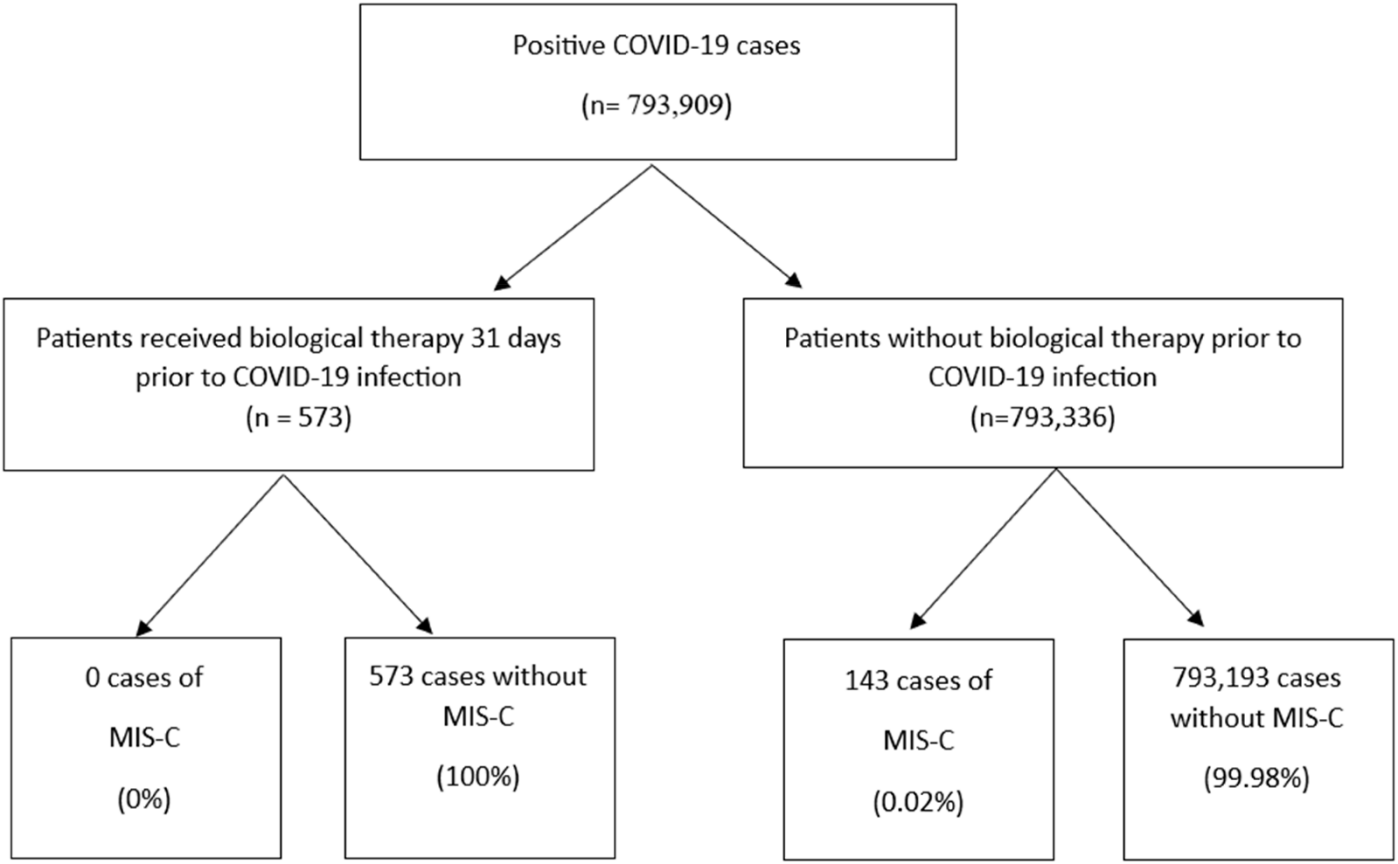
The process of patient selection and inclusion.

This study examined data from 793,909 cases aged 0–18 who tested positive for COVID-19, of whom 143 (0.02%) developed MIS-C. The median age at the time of COVID-19 infection was slightly lower in the MIS-C group compared to those without MIS-C [7.93 years (IQR 4.85–10.60) vs. 9.27 years (IQR 5.52–13.04), *p* = 0.2]. Biologic and targeted-synthetic therapies were administered to only 0.1% of the total cohort (*n* = 573), all within the non-MIS-C group (Table 1). The process of patient selection and inclusion is pictorially shown in Figure 1.

**Table 1:** Comparison between patients without MIS-C and patients with MIS-C.

Characteristic	Patients without MIS-C (*n* = 793,766)	Patients with MIS-C (*n* = 143)	*P*-value
Patients not receiving biologic and targeted-synthetic therapies	793,192 (99.9%)	143 (100%)	
Patients receiving biologic and targeted-synthetic therapies	573 (0.1%)	0 (0%)	
Age at covid infection, median (IQR)	9.27 (5.52, 13.04)	7.93 (4.85,10.60)	0.2
Gender (Male), *n* (%)	3,97,626 (50%)	82 (57%)	0.10

### Patients treated with biologic and targeted-synthetic therapies

Regarding the 573 patients treated with biologic and targeted-synthetic therapies; the average duration between biologic and targeted-synthetic therapies administration and the positive COVID-19 test was 16 ± 9.2 days. The most prevalent biological drug used was Adalimumab, in 236 (41.2%) patients, followed by Infliximab in 174 (30.4%) patients; both are anti-TNF α drugs. Etanercept, another anti-TNF *α* drug, was used in 35 (6.1%) of the patients, making anti-TNF α drugs the most used drugs in our cohort. Table 2 presents the prevalence of the biologic and targeted-synthetic therapies used for treatment in this cohort. Additionally, regarding the medical conditions that required treatment with biological or targeted-synthetic therapies, some patients had more than one diagnosis. Unfortunately, most of the patients had no obvious diagnosis warranting treatment with biological therapies according to the medical records. Among the known diagnoses, Inflammatory Bowel Disease (IBD) was the most prevalent, affecting 189 (15%) of the patients, followed by Juvenile Idiopathic Arthritis (JIA), which affected 93 (7.2%) of the patients. Table 3 presents all the medical conditions and their prevalences.

**Table 2:** Biologic and targeted-synthetic therapies.

Biological Drug	Number of patients (%)
Adalimumab	236 (41.2%)
Infliximab	174 (30.4%)
Canakinumab	62 (10.8%)
Etanercept	35 (6.1%)
Rituximab	21 (3.7%)
Ustekinumab	17 (3%)
Tocilizumab	13 (2.3%)
Tofacitinib	6 (1%)
Abatacept	4 (0.7%)
Anakinra	3 (0.5%)
Golimumab	2 (0.3%)

**Table 3:** Medical condition treated with biologic and targeted-synthetic therapies.

Medical condition	*N* = 1,283^a^
Unknown	877 (68.3%)
IBD	189 (15%)
JIA	93 (7.2%)
Psoriasis	42 (3.3%)
FMF	34 (2.7%)
Hidradenitis Suppurativa	26 (2.0%)
Optic Neuritis	7 (0.5%)
Behçet's disease	6 (0.5%)
Rheumatic/Autoimmune	5 (0.4%)
CRMO	1 (<0.1%)
SLE	1 (<0.1%)
Synovitis And Tenosynovitis	1 (<0.1%)
Takayasu arteritis	1 (<0.1%)

CRMO, chronic recurrent multifocal osteomyelitis; FMF, familial mediterranean fever; IBD, inflammatory bowel disease; JIA, Juvenile idiopathic arthritis; SLE, systemic lupus erythematous.

^a^ The number of diagnoses exceeds the number of patients as some individuals had multiple medical conditions.

### MIS-C cases

MIS-C was identified in 143 children of the whole cohort (18 cases per 100,000 participants) between 28 and 42 days after their positive COVID-19 test. The average duration from the positive COVID-19 test to MIS-C diagnosis was 40.8 ± 3.2 days. The patients diagnosed with MIS-C had a median age of 7.93 (IQR: 4.85,10.60), with 57% being males (Table 1). Remarkably, none of the participants who received biologic and targeted-synthetic therapies were diagnosed with MIS-C.

Among 793,335 patients who were not treated with biologic and targeted-synthetic therapies, 143 MIS-C cases were detected (risk = 0.00018). In contrast, no MIS-C cases were recorded among the 573 individuals who received biologic and targeted-synthetic therapies. The calculated relative risk (RR) was 0, indicating no observed association between biological treatment status and the occurrence of MIS-C. The uncorrected odds ratio was also 0 [95% CI: (0, 0.38)], and Fisher’s exact test returned a non-significant *p*-value (*p* = 1). These counterintuitive results reflect the extremely low frequency of MIS-C cases overall and the conservative nature of the Fisher test when applied to highly unbalanced tables with small expected counts. Despite the non-significant *p*-value from Fisher’s test, and the 0 RR and OR, the absence of events in the biologic treatment group suggests a clinically relevant reduction in risk.

## Discussion

MIS-C presents a significant challenge in the context of the COVID-19 pandemic due to its potentially severe clinical manifestations, including significant cardiac involvement and a mortality rate of approximately 2% (4). Understanding the factors that may influence the development of MIS-C is crucial for guiding clinical management and improving patient outcomes.

Biological therapies have emerged as a potential treatment option for MIS-C, targeting the dysregulated proinflammatory cytokines implicated in its pathogenesis (5). However, the relationship between biological therapy and the risk of developing MIS-C in the context of COVID-19 infection remains unclear. This study aimed to address this gap by comparing the incidence of MIS-C among individuals treated with biologic and targeted-synthetic therapies vs. those who did not receive such treatments.

In this “big data” study, none of the children who received biologic and targeted-synthetic therapies were diagnosed with MIS-C, indicating a potential protective effect. Although Fisher’s exact test and logistic regression analysis were not applicable due to the absence of MIS-C cases among patients treated with biologic and targeted-synthetic therapies, the findings of this study suggest that biologic and targeted-synthetic therapies may indeed play a role in mitigating the risk of MIS-C following COVID-19 infection in children despite the inability of statistical tests to demonstrate this mathematically due to the lack of observed cases. Given that MIS-C is rare, affecting only 0.02% of patients not receiving biologic and targeted-synthetic therapies, statistical proof of this protective effect remains challenging. Nonetheless, the lack of MIS-C cases in the biological therapy group supports this as a reasonable assumption.

Our results align with a previous study involving a cohort of 31 patients previously treated with biological therapies, which reported zero cases of MIS-C in this group (11). However, they contradict two other studies involving cohorts of 26 (12) and 113 (13) patients, which revealed a percentage of 4.4% to 19.2% of MIS-C development in patients treated with biological drugs. Based on a large number of patients treated with biological drugs from multiple medical centers across the country, our study is less susceptible to selection biases that often affect single-center studies. In addition, it contributes significantly to the existing data and may influence the understanding of MIS-C occurrence in patients treated with biological drugs, potentially tilting the scale towards a zero percentage.

In addition, these findings contribute to the growing body of literature on the management of MIS-C and underscore the importance of considering biologic and targeted-synthetic therapies as a potential therapeutic option in this population (14).

The potential protective effect of biological therapies in preventing MIS-C may be linked to their ability to modulate the immune response, particularly through the regulation of cytokine storms and inflammation. Biological therapies, such as TNF inhibitors, IL-6 inhibitors, and other targeted agents, are known to interfere with the dysregulated immune signaling that often occurs in severe COVID-19 and related complications.

Understanding the immune responses in inflammatory conditions is crucial for elucidating the pathogenesis of MIS-C. For instance, disturbances in the interaction between gut-resident macrophages and the gut microbiota can lead to IBD (15). The dysregulated immune responses observed in IBD may share similarities with those in MIS-C, suggesting that alterations in macrophage function could contribute to the development of MIS-C. Additionally, oxidative stress can lead to the depletion of tetrahydrobiopterin (BH4), a critical cofactor in nitric oxide synthesis, impairing endothelial function (16). Given that endothelial dysfunction is a hallmark of MIS-C, these insights highlight the importance of redox balance in the pathogenesis of MIS-C. Furthermore, the plasticity of immune cells, particularly macrophages, allows for a dynamic response to inflammatory stimuli (17). This adaptability may influence the severity and progression of inflammatory conditions, including MIS-C, underscoring the need for targeted therapeutic strategies that modulate immune cell function.

In the case of MIS-C, a hyper-inflammatory response, characterized by an overproduction of pro-inflammatory cytokines, plays a crucial role in the development of the syndrome. By targeting these inflammatory pathways, biologic and targeted-synthetic therapies may help reduce excessive immune activation, thereby potentially preventing the onset of MIS-C. However, further research is needed to fully elucidate the exact mechanisms through which these therapies influence the progression of MIS-C and confirm their therapeutic potential in this context.

Several limitations should be acknowledged. One of the major limitations of this study is the low number of MIS-C events, due to the rarity of the disease, which may affect the statistical power and reliability of the findings. An additional significant limitation is the lack of data on the clinical reasons for initiating biologic and targeted-synthetic therapies in most of the children treated. This absence of indication data limits our ability to fully interpret the results and increases the potential for residual confounding. Furthermore, the retrospective design and the data structure of this large-scale, real-world dataset prevented a more refined analysis of pharmacokinetics and the specific dosages of biologic and targeted-synthetic therapies used, and whether other anti-inflammatory or immunomodulatory treatments were administered simultaneously, which may influence their effectiveness in preventing MIS-C. Finally, the study population was limited to children aged 0–18, and individuals with underlying comorbidities were excluded, which limits the generalizability of the findings.

## Conclusion

The results of this study suggest a possible association between biological and targeted-synthetic therapies and reduced risk of MISC following COVID-19 infection in children. However, due to the observational nature of the study, further research is needed to confirm these observations and establish causality. Prospective studies with larger sample sizes and longer follow-up periods are essential to provide more definitive evidence and better inform clinical practice regarding the optimal management of MIS-C in pediatric patients with COVID-19.

## Data Availability

The raw data supporting the conclusions of this article will be made available by the authors, without undue reservation.
